# Changes in the Q-tip angle in relation to the patient position and bladder filling

**DOI:** 10.1186/s12894-015-0096-4

**Published:** 2015-10-07

**Authors:** Jong-hyun Yun, Jae Heon Kim, Suyeon Park, Changho Lee

**Affiliations:** Department of Urology, Soonchunhyang University Gumi Hospital, Soonchunhyang University School of Medicine, Gumi, South Korea; Department of Urology, Soonchunhyang University Hospital, Soonchunhyang University School of Medicine, Seoul, South Korea; Department of Biostatistics, Soonchunhyang University Hospital, Seoul, South Korea; Department of Urology, Soonchunhyang University Cheonan Hospital, Soonchunhyang University School of Medicine, 31 Soonchunhyang 6 gil, Dongnam-Gu, Cheonan, Chungcheongnam-do 330-721 South Korea

**Keywords:** Q-tip test, Stress urinary incontinence, Urethra, Urinary incontinence

## Abstract

**Background:**

It is hypothesized that patient position, supine or recline, and bladder filling status, empty or full, could change the Q-tip test result. This study evaluated the effect of the patient position and bladder filling status on the Q-tip angle for urethral hypermobility (UH).

**Methods:**

There was a measurement of the Q-tip angle in the supine position and at a 45° angle in a reclining position during bladder emptying; and then the measurements were repeated while filling the bladder. We defined urethral hypermobility as the urethral angle straining or coughing minus that at rest ≥30°.

**Results:**

All 63 female patients (mean age: 61.6 years, range: 36–81) who complained of urinary incontinence were assessed using the Q-tip angle test. The pelvic organ prolapse quantification stages of all patients were ≤ stage 1. The mean Q-tip angle with an empty bladder was 14.1 ± 9.1° in the supine position and 16.4 ± 11.1° in the reclining position (*p* = 0.001). Then mean Q-tip angle during the filling bladder state was 15.4 ± 9.7° in the supine position and 15.9 ± 11.0° in the reclining position (*p* = 0.771). The UH rate during the bladder emptying state was 11.1 % (7/63) in the supine position and 19.1 % (12/63) in the reclining position. The UH rate during the bladder filling state was 15.0 % (9/60) in the supine position and 15.3 % (9/59) in the 45° reclining position. The odds ratio (OR) was 7.03 in the reclining position for a positive Q-tip angle. The positive rate was higher in the 45° reclining position during bladder emptying than that in the other position during bladder filling.

**Conclusion:**

The outcome of the Q-tip angle and the rate of UH changed in relation to patient position. The reclining position during bladder emptying increased the Q-tip angle, thereby resulting in a positive UH.

## Background

Urinary incontinence (UI) is the involuntary loss of urine and occurs when bladder pressure exceeds urethral closing pressure. A specific type of UI is stress urinary incontinence (SUI) is a complaint of involuntary urine leakage on effort or exertion, or on sneezing or coughing [1].

A poorly supported bladder base and urethrovesical junction (UVJ) are the main explanations for SUI; thus, urethral mobility should be assessed in all women with UI [2, 3]. The cotton swab or Q-tip test is a simple outpatient procedure used to quantify urethral mobility [4]. Although the Q-tip test was introduced more than 40 years ago, and is widely used in clinical practice, there is limited literature on its use. Furthermore, only a few studies have evaluated the Q-tip examination based on patient position and bladder filling status [5, 6].

We hypothesized that patient position (supine or recline), and bladder filling status (empty or full), would change the Q-tip test result, as patient position affects bladder pressure, and bladder filling status can change the shape of the bladder base. To verify our hypothesis, we evaluated the effects of patient position and bladder filling status of Q-tip test results for urethral hypermobility (UH).

## Methods

We reviewed the medical records of women who presented to one urologist (CL) with urinary incontinence between February 2010 and March 2014. The clinical diagnosis was established based on history taking, in particular, the chief complaints of patients. All patients underwent a pelvic examination, including a pelvic organ prolapse quantification (POP-Q) measurement and a Q-tip test performed by the same urologist (CL) in the dorsal lithotomy position. The urologist first performed the POP-Q test in the supine dorsal lithotomy position and then performed a Q-tip test. The Q-tip test was performed four times in each patient under different conditions. A sterile rubber Nelaton catheter was inserted into the bladder for emptying. Then, a sterile lubricated cotton swab (Q-tip) was introduced through the urethra into the bladder, and then withdrawn carefully until a resistance could be felt. This was considered the anatomical location of UVJ, with the length of inserted Q-tip is between 3.5 and 4.5 cm from the urethral meatus in all evaluated patients. The resting angle from the horizontal was set to 0° on a goniometer. The patient was asked to strain or cough while the maximum straining angle was measured and recorded as the Q-tip angle. This UVJ angle measurement was done in the supine position and is deemed the first measured Q-tip value. For the second Q-tip measurement value, the patient was moved to a reclining position (approximately 45°) by setting the backrest up on the examination table, and the same Q-tip measurement was repeated. Another sterile rubber Nelaton catheter was inserted, and normal saline was slowly infused using a Toomey syringe until the patient expressed a desire to void. The mean infused volume is 209.7 ± 59.4 mL (range: 50–400) and there is no significant difference of bladder filling volume between 31 MUI and 3 UUI patients versus 27 SUI patients (mean bladder filling volume 209.4 ± 68.4 vs. 210.0 ± 47.2, *p* = 0.22). The third and fourth Q-tip values for the Q-tip angle was again a measurement in the supine and reclining position. The incontinence provocation test was performed at the end of physical examination, a stress test using coughing. A positive result is indicated by efflux of the bladder solution from the meatus coinciding with the cough.

We reviewed the four Q-tip values and provocation test results on the medical records. There was an exclusion of the Q-tip angle data for patients with prolapsed pelvic organs, those over stage 1 according to the POP-Q criteria, and patients who had undergone surgery to correct incontinence. Urethral hypermobility was defined as the urethral angle straining or coughing minus that at rest ≥30°.

The statistical analysis was performed using SPSS ver. 18.0 software (SPSS Inc., Chicago, IL, USA). The paired *t*-test was used to evaluate the Q-tip angles associated with the patients’ positions, and those associated with bladder filling status. The generalized linear mixed model was used to calculate the odds ratio and confidence interval. A *p*-value <0.05 was considered significant.

### Ethics statement

This study protocol was approved by the Soonchunhyang University institutional review board of the human research and ethics committee (No. 1040875-201501-BM-002). They waived the requirement for the investigator to obtain a signed consent form.

## Results

### Patients

We reviewed all 75 medical records of women with urinary incontinence. Among them, we excluded the following: 4 records with pelvic organ prolapsed greater than stage 1; 2 records of previous anti-incontinence surgery; 3 records of performing evaluation two times at different visits; and 3 records of a negative Q-tip value. A total of 63 patients complaining of UI were assessed. Among them, 32 (50.8 %) had mixed urinary incontinence (MUI), 27 (42.9 %) had stress urinary incontinence (SUI), and four (6.3 %) had urgency urinary incontinence (UUI).

Among the 63 patients, 58 had provocation test results; 55.2 % (32/58) had positive provocation test results. The patients with positive provocation test results had more hypermobile urethra than the patients with negative provocation test results (Table 1). Of the 63 patients with UI, 25 (39.7 %) received a mid-urethral sling (MUS) to manage SUI. Among the 25 patients with MUS, 24 had the following Valsalva leak point pressure (VLPP) data; 33.3 % (8/24) had VLPP ≤60 cm H_2_O, 41.7 % (10/24) had VLPP of 60–89 cm H_2_O, and 25.0 % (6/24) had VLPP ≥90 cm H_2_O. The patients with low VLPP had a tendency of more mobile urethra (Table 1).

**Table 1:** Basic characteristics of the patients (*n* = 63)

All patients (*n* = 63)			
	Age (range), years	61.6 ± 11.1 (36–81)	
	Clinical Diagnosis, n		
	Mixed urinary incontinence	32/63 (50.8 %)	
	Stress urinary incontinence	27/63 (42.9 %)	
	Urgency urinary incontinence	4/63 (6.3 %)	
			Hypermobile Urethra (Q tip ≥30)
	Provocation test positive, n	32/58 (55.2 %)	8/32 (25.0 %)
	Provocation test negative, n	26/58 (44.8 %)	3/26 (11.5 %)
MUS patients (*n* = 25)			
	Age (range), years	61.6 ± 11.0 (46–81)	
	Clinical Diagnosis, n		
	Mixed urinary incontinence	12/25 (48.0 %)	
	Stress urinary incontinence	13/25 (52.0 %)	
	BMI (kg/m^2^)	25.6 ± 2.9	
	Post-hysterectomy status	3/25 (12.0 %)	
	Smoking	0/25 (0.0 %)	
			Hypermobile Urethra (Q tip ≥30)
	VLPP ≤60	8/24 (33.3 %)	50.0 % (4/8)
	VLPP 61–89	10/24 (41.7 %)	40.0 % (4/10)
	VLPP ≥90	6/24 (25.0 %)	16.7 % (1/6)

### Q-tip angle change in relation to position

We analyzed the Q-tip angle change in the 63 patients complaining of UI. The mean Q-tip angle during the bladder emptying state was 14.1 ± 9.1° in the supine position and 16.4 ± 11.1° in the reclining position (*p* = 0.001). The mean Q-tip angle during the bladder filling state was 15.4 ± 9.7° in the supine position, and 15.9 ± 11.0° in the reclining position (*p* = 0.771) (Table 2).

**Table 2:** Comparison of mean Q-tip angles and urethral hypermobility (UH) rates in relation to patient position

	Position	Average Q-tip	*p*-value†	Q-tip ≥30°	Odds ratio	95 % CI (Confidence interval)	*p*-value††
Empty	Supine	14.1 ± 9.1	0.001	11.1 % (7/63)	7.03^a^	1.01–48.94	0.05
	Reclining	16.4 ± 11.1		19.1 % (12/63)			
Filling	Supine	15.4 ± 9.7	0.771	15.0 % (9/60)			
	Reclining	15.9 ± 11.0		15.3 % (9/59)			

^a^Reclining position had higher odds of a positive Q-tip angle, *p*-value†; by paired *t*-test, *p*-value††; by generalized linear mixed model

**Fig. 1:**
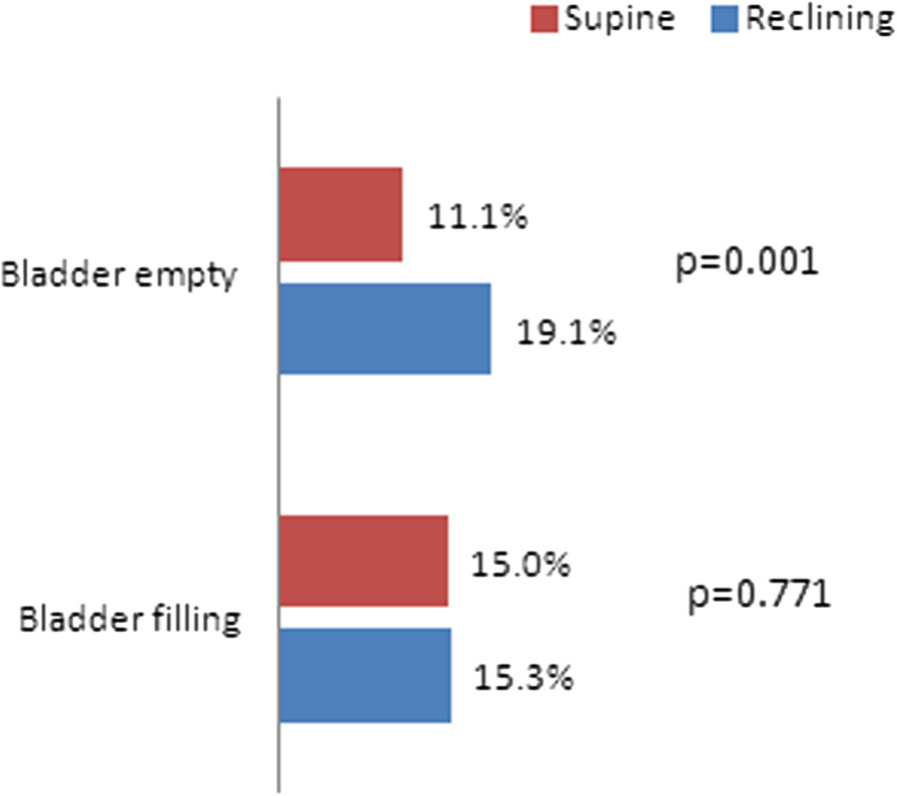
Comparison of the urethral hypermobility rate in relation to patient position. Positive urethral hypermobility was defined as a Q-tip angle ≥30°

The proportion of patients identified with UH during the bladder emptying state (Q-tip angle ≥30°) was 11.1 % (7/63) in the supine position and 19.1 % (12/63) in the reclining position. The proportion of patients with UH during the bladder filling state was 15.0 % (9/60) in the supine position and 15.3 % (9/59) in the reclining position. The reclining position had an odds ratio of 7.03 for a positive Q-tip angle (Table 2 and Fig. 1).

### Q-tip angle change in relation to bladder filling state

The mean Q-tip angle was 14.1 ± 9.1° in the supine position during the bladder emptying state and 15.4 ± 9.7° during the bladder filling state (*p* = 0.049). The mean Q-tip angle was 16.4 ± 11.1° in the reclining position during the bladder emptying state and 15.9 ± 11.0° during the bladder filling state (*p* = 0.361) (Table 3).

**Table 3:** Comparison of mean Q-tip angles and urethral hypermobility (UH) rates in relation to bladder filling status

	Position	Average Q-tip	*p*-value†	Q-tip ≥ 30°	Odds Ratio	95 % CI (Confidence Interval)	*p*-value††
Supine	Empty	14.1 ± 9.1	0.049	11.1 % (7/63)	1.45^a^	0.25–8.36	0.67
	Filling	15.4 ± 9.7		15.0 % (9/60)			
Reclining	Empty	16.4 ± 11.1	0.361	19.1 % (12/63)			
	Filling	15.9 ± 11.0		14.3 % (9/63)			

^a^Bladder filling status had higher odds of a positive Q-tip angle, *p*-value†; by paired *t*-test, *p*-value††; by generalized linear mixed model

**Fig. 2:**
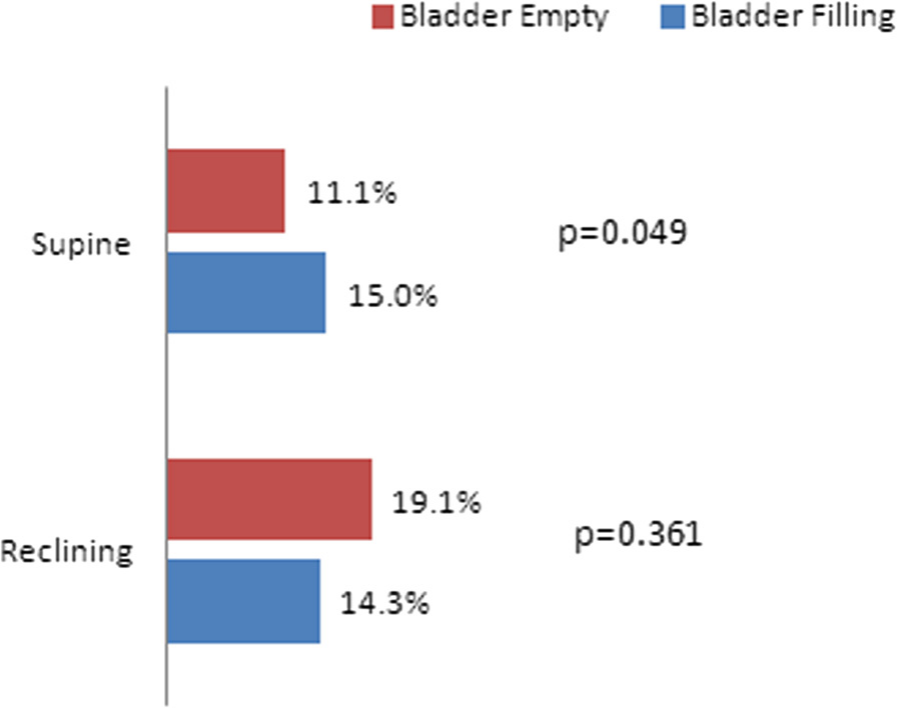
Comparison of the urethral hypermobility rate in relation to bladder filling status. Positive urethral hypermobility was defined as a Q-tip angle ≥30°

The proportion of patients defined as UH during the bladder emptying state in the supine position (Q-tip angle ≥30°) was 11.1 % (7/63) and was 15.0 % (9/60) during the bladder filling state. The proportion of patients identified with UH in the reclining position was 19.1 % (12/63) during the bladder emptying state and 14.3 % (9/63) during the bladder filling state. The bladder filling status had an odds ratio of 1.45 for a positive Q-tip angle (Table 3 and Fig. 2).

## Discussion

The Q-tip test was developed to measure the degree of urethral relaxation and mobility during increased intra-abdominal pressure [4, 7, 8]. When a physician performs the Q-tip test, patients are usually positioned in the supine lithotomy position, then the Q-tip is inserted into the bladder through the urethra, and the angle that the Q-tip moves from horizontal to its final position during straining is measured [5, 8]. The UH was initially defined as a Q-tip angle ≥ 20° from the horizontal position [4]. However, a Q-tip angle ≥30° appears to be widely accepted by urologists and urogynecologists [3, 9].

The Q-tip angle measures the degree of urethral mobility presented by increased intra-abdominal pressure. Abdominal pressure is approximately two times higher with the patient in the seated position than that when the patient is in the supine position [10]. Because the patient position affects abdominal pressure, we hypothesized that the patient position would also affect the Q-tip angle measurement. In this study, the mean Q-tip angle was 14.1 ± 9.1° in the supine position during the empty bladder state and 16.4 ± 11.1° in the reclining position (*p* = 0.001, Table 2). We calculated the UH rate by using a 30° cut-off value to clarify whether this difference was clinically significant. The UH rate was 11.1 % in the supine position in the bladder emptying state, and 19.1 % in the reclining position. The odds ratio was 7.03 in reclining position (Table 2 and Fig. 1). Therefore, we concluded that the patient position (supine or reclining) significantly affected the change in Q-tip angle. The female urethra is more mobile in the reclining position than in the supine position because of elevated abdominal pressure in the reclining position.

The patient’s bladder filling status is usually not considered when physicians measure the Q-tip angle and no reliable recommendations are available on bladder filling status while measuring the Q-tip angle [3–5, 7–9]. We hypothesized that the bladder filling status would affect the Q-tip angle measurement because it changes the shape of bladder base. Only one report has analyzed Q-tip angle measurements in patients with a symptomatically full bladder [5]. They reported no difference in either resting or straining Q-tip angle measurements when patients were tested with <150 mL of urine in their bladder as compared with patients with a symptomatically full bladder. We emptied the patient’s bladder completely using a Nelaton catheter and measured the Q-tip angle in the supine and reclining positions. Then, we filled the patient’s bladder until they expressed a desire to urinate, and measured the Q-tip angle again in the supine and reclining positions. The odds ratio was 1.45 for the filled bladder and there is no significant difference in the Q-tip angles between empty and full bladders (Table 3 and Fig. 2). Although these findings are supported by previous studies, the reasons are not well understood as to why bladder filling status does not affect the Q-tip angle. Moreover, although we found that the reclining position increased the Q-tip angle, this effect dissipated when we measured the Q-tip angle during the bladder filling state. We anticipated that some patients would not have an active response to straining or coughing because of worry or shyness about leaking. We believe that this may be a habitual coping method by some incontinent patients, which may explain why some patients did not show consistent Q-tip angle measurements during the bladder filling state.

Yet, an important question is: which position and bladder filling condition should be recommended for detecting urethral hypermobility? A good physical examination is a reflection of a patient’s complaints or condition. Most incontinence events in patients with SUI occur in the standing position. Thus, the standing position might be the best position for the Q-tip test. However, previous study showed that the urethra is more mobile in the supine position than that in the standing position, and the sitting position results in a more mobile urethra than that of the supine position [6, 11]. Our current results also demonstrate that the reclining produces a more mobile urethra than that of the supine position (Table 2 and Fig. 1). A more mobile urethra has decreased Valsalva leak point pressure and is associated with the success rate of the tension-free vaginal tape procedure [12, 13]. These observations indicate that UH is an explanation for SUI but not for the female SUI mechanism.

Our study has some limitations, of which the first is its retrospective nature, which did not ensure complete data collection for all patients. Among enrolled 63 patients, we could not obtain important variables, such as body mass index (BMI), parity, and the post-hysterectomy status, except for 25 patients who had undergone an MUS. Moreover, this current study did not answer important question as to which measurement correlated with a better surgical outcome. However, the study population in this study of having an MUS is too small to answer that important question. Thus that question will have to be answered by further research.

In summary, we do not know which position more closely reflects urethral mobility in patients with SUI. The reclining position guaranteed the most mobile urethra, whereas the supine position provided a more mobile urethra than that of the standing position. We recommend an empty bladder or urine volume less than the volume when voiding is desired. According to a previous report and our result, the bladder filling status does not affect UH [5]. However, an empty bladder or a bladder with a small volume of urine was more comfortable for the examinees.

## Conclusion

The outcome of Q-tip angle measurement and the rate of UH appeared to increase when patients were examined in the reclining position. However, this difference dissipated when the Q-tip angle was measured during the bladder filling state. The largest difference in Q-tip angle, indicating a positive UH, was observed in patients in the reclining position during the bladder emptying state.

## Abbreviations

UH: Urethral hypermobility
UI: Urinary incontinence
SUI: Stress urinary incontinence
UVJ: Urethrovesical junction
POP-Q: Pelvic organ prolapsed quantification
MUI: Mixed urinary incontinence
UUI: Urgency urinary incontinence
MUS: Mid-urethral sling
VLPP: Valsalva leak point pressure
